# Supplementary material for: MetaRibo-Seq measures translation in microbiomes
Source: Nat Commun. 2020 Jun 29;11:3268. doi: 10.1038/s41467-020-17081-z (PMC7324362; doi:10.1038/s41467-020-17081-z)
Supplement: Supplementary file 10 — Supplementary Data 7 [file 41467_2020_17081_MOESM10_ESM.zip › File2/Confidence_High_Taxonomy/133951_out.krona.html]

Javascript must be enabled to view this page.

members
magnitude
magnitudeUnassigned
count
unassigned
taxon
rank

133951\_out

5

2759
2
superkingdom

4751
2
kingdom

451864
subkingdom
2

2
phylum
4890

1
147538

SRS023914\_contig\_number\_9535
subphylum
2

147547
class
1

388435
1
subclass

1
order
107465

1
suborder
1302176

1
family
88646

1301213
1
subfamily

genus
1
1300028

species
1

SRS1041145\_contig\_number\_3317
1301419

2
superkingdom
3

phylum
3
201174

3
class

SRS013687\_contig\_number\_27828
1760
1

order
2
85011

family
2
2062

1883
2
genus

species group
2
629295

1482596
2
species subgroup


SRS051031\_contig\_number\_36149SRS076929\_contig\_number\_contig-100\_774.148600
2
species
1911
